# The PARP inhibitor Olaparib disrupts base excision repair of 5-aza-2′-deoxycytidine lesions

**DOI:** 10.1093/nar/gku638

**Published:** 2014-07-29

**Authors:** Manuel Luis Orta, Andreas Höglund, José Manuel Calderón-Montaño, Inmaculada Domínguez, Estefanía Burgos-Morón, Torkild Visnes, Nuria Pastor, Cecilia Ström, Miguel López-lázaro, Thomas Helleday

**Affiliations:** 1 Department of Cell Biology, Faculty of Biology, University of Seville, Avda. Reina Mercedes 6, 41012 Seville, Spain; 2 Science for Life Laboratory, Division of Translational Medicine and Chemical Biology, Department of Medical Biochemistry and Biophysics, Karolinska Institute, S-171 21 Stockholm, Sweden; 3 Department of Pharmacology, Faculty of Pharmacy, University of Seville, c/Professor García González, No. 2, 41012 Seville, Spain

## Abstract

Decitabine (5-aza-2′-deoxycytidine, 5-azadC) is used in the treatment of Myelodysplatic syndrome (MDS) and Acute Myeloid Leukemia (AML). Its mechanism of action is thought to involve reactivation of genes implicated in differentiation and transformation, as well as induction of DNA damage by trapping DNA methyltranferases (DNMT) to DNA. We demonstrate for the first time that base excision repair (BER) recognizes 5-azadC-induced lesions in DNA and mediates repair. We find that BER (XRCC1) deficient cells are sensitive to 5-azadC and display an increased amount of DNA single- and double-strand breaks. The XRCC1 protein co-localizes with DNMT1 foci after 5-azadC treatment, suggesting a novel and specific role of XRCC1 in the repair of trapped DNMT1. 5-azadC-induced DNMT foci persist in XRCC1 defective cells, demonstrating a role for XRCC1 in repair of 5-azadC-induced DNA lesions. Poly (ADP-ribose) polymerase (PARP) inhibition prevents XRCC1 relocation to DNA damage sites, disrupts XRCC1–DNMT1 co-localization and thereby efficient BER. In a panel of AML cell lines, combining 5-azadC and Olaparib cause synthetic lethality. These data suggest that PARP inhibitors can be used in combination with 5-azadC to improve treatment of MDS and AML.

## INTRODUCTION

5-aza-2′-deoxycytidine (5-azadC) and 5-azacytidine (5-azaC) are synthetic cytidine analogues highly effective in the treatment of myelodysplastic syndromes (MDS), with 5-azadC also showing good clinical response in older patients diagnosed with acute myeloid leukemia (AML) (1–3). 5-azaC and 5-azadC were synthesized in the 60s as conventional cytostatic drugs (4) but were later discovered to demethylate DNA through their interactions with DNA methyltransferases (DNMTs) (5).

It is well accepted that the antineoplastic properties of these agents are due to two non-exclusive mechanisms. First, demethylation causes reactivation of hypermethylated/silenced tumor suppressor genes and second, DNMT–DNA adducts causes genome wide DNA damage (6–8).

It has been suggested that the base excision repair pathway (BER) could be involved in the excision of 5-azaC and 5-azadC from DNA (9). The BER pathway is initiated by DNA glycosylases that recognize and excise aberrant bases from DNA, generating an abasic (AP) site in DNA, further processed by AP-endonuclease 1 (APE 1) to form a single-strand break (SSB) in DNA. A DNA polymerase replaces the missing nucleotide and a complex constituted by DNA ligase and the X-ray repair cross-complementing protein 1 (XRCC1) finalizes repair by ligating DNA (10).

XRCC1 is well known to be essential for both BER and SSB repair. XRCC1 deficient mice are embryonic lethal (11) and XRCC1 deficient cells display increased levels of spontaneous γ-H2AX and RAD51 foci (12), and are hypersensitive to agents that induce SSBs or base damage (13,14).

In this paper we demonstrate for the first time that BER is required to repair DNA lesions induced by 5-azadC. BER (XRCC1) deficient cells displayed reduced survival and increased levels of single- and double-strand breaks (DSBs) as well as chromosomal abnormalities. Our findings suggest a novel and specific role of XRCC1 in the repair of DNA damage and survival of cancer cells following 5-azadC treatment. We and others have previously shown that Olaparib treatment traps PARP on the single strand DNA break intermediate generated during BER and prevents further repair (15,16). In this paper we show that 5-azadC in combination with the PARP inhibitor Olaparib blocks BER induced by 5-azadC and leads to a synergistic induction of cell death in a panel of AML cells. We believe that this combination treatment warrants further investigation in the hope that this could improve current best supportive care of MDS and AML patients.

## MATERIALS AND METHODS

### Chemicals and plasmids

5-aza-2′-deoxycytidine and 5-azacytidine (Sigma) were dissolved in phosphate buffered saline (PBS) and stored at −80ºC. The plasmid expressing GFP-tagged DNMT1 was a gift from Keith Robertson and the RFP-tagged XRCC1 plasmid was a gift from Heinrich Leonhard, both described previously (6,17). Olaparib (KU-0059436, AZD-2281) and 4-amino 1,8 naphthalimide (4-ANI) were dissolved in DMSO, aliquoted and stored at −80ºC. Methoxyamine (MX) was purchased from Sigma and freshly dissolved in PBS before use.

### Cell lines and culture conditions

Chinese hamster ovary cells AA8 (wild-type) and EM9 (XRCC1 deficient) were cultured in McCoy's 5A media. EM9 cells stably transfected with an empty vector (EM9-V) or with a vector encoding human *XRCC1* (EM9-XH) were a gift from Keith Caldecott and have been described earlier (18). These cells were cultured in Dulbecco's modified Eagle's medium (DMEM), in the presence of geneticin (G418) at a final concentration of 1.5 mg/ml.

The AML cell line HL60 was obtained from ATCC, whereas K652, KG1a, Mv4–11 and PL21 cells were kindly provided by Dr Sören Lehmann, Karolinska Institutet, Sweden. All AML cells were grown in RPMI 1640-Glutamax. HeLa cells were obtained from ATCC and grown in DMEM.

All cell lines were cultured with 10% fetal calf serum, penicillin (50 U/ml) and streptomycin (50 μg/ml), at 37°C in a 5% CO_2_ atmosphere.

### Survival assay

EM9-XH and EM9-V cells were seeded in 10 cm Petri dishes 4 h prior to 5-azadC or 5-azaC treatment for 24 h. Colonies were fixed, stained (methylene blue dissolved in methanol, 4 g/l) and counted after 7–10 days. The data are plotted as percentage of survival referred to control cells. Procedures for combination experiments are further described in figure legends.

### Cytogenetic spreads and DNA damage scoring

AA8 and EM9 cells were treated with 5-azadC for 24 h, washed and kept in fresh medium for 12 h to recover. Chromosome spreads were prepared as described previously (7).

### Immunofluorescence

The cells were treated for 24 h with 5-azadC, washed and incubated for 30 s with ice cold 0.1% Triton-X in PBS to pre-extract soluble protein and then fixed in 4% paraformaldehyde at room temperature for 10 min. Inmunofluorescence was carried out as previously described (7).

Cells with more than 10 or 20 foci were counted as positive for RAD51 or γ-H2AX respectively. At least 200 nuclei were counted on each slide.

For γ-H2AX and 53Bp1 quantification in AML HL60 cells, following 24 h exposure to 100 nM 5-azadC and/or 1 μM Olaparib, cells were washed with PBS and incubated for 20 min with cold methanol. Cells were washed again with PBS and incubated for 10 min with 5% bovine serum albumin (BSA), 0.1% Tween-20 and 0.1% Saponin in PBS. All subsequent steps were performed with 0.1% Saponin in the media. Cells were then incubated overnight at 4°C with primary antibodies, anti-phospho-histone H2AX (S139; 1:1000; Millipore) and anti-53Bp1 (1:1000; Bethyl). The cells were then washed and incubated with secondary antibodies goat anti-mouse IgG Alexa Fluor^®^ 488 (1:1000, Life technologies) and donkey anti-rabbit IgG IgG Alexa Fluor^®^ 555 (1:1000, Life technologies). DNA was stained with 100 nM 4,6 diamidino-2-phenylindole for 15 min. Finally, cells were attached to microscope slides by centrifugation for 4 min at 600 rpm in a cytospin centrifuge and processed for confocal microscope analysis. A total of 200 cells/slide were counted and cells with 10 or more co-localized foci of phospho-histone H2AX and 53BP1 were scored as positive.

### Alkaline DNA unwinding technique experiments

Cells were treated with 50 μM 5-azadC for 24 h together with [^3^H]-TdR (7.1 kBq/ml), then lysed at different time points and processed for the alkaline DNA unwinding (ADU) technique to quantify DNA breaks.

In order to monitor the effect of PARP inhibition during 5-azadC treatment, cells were cultured in the presence of PARP inhibitor Olaparib (1 μM) during the last 12 h of treatment. The ADU technique was performed as previously described (15,19–21).

### Transfection and co-localization procedures

All transient transfections were performed using JetPRIME^®^ transfection reagent according to the manufacturer's protocol (PolyPlus).

HeLa cells were transfected with DNMT1-GFP and XRCC1-RFP plasmids and then treated with 5-azadC (15 μM) alone or in the presence of Olaparib (1 μM) for 24 h. The cells were fixed with 4% paraformaldehyde and processed for confocal microscope analysis. Manders co-localization index was measured using Fiji software (Coloc2 pluggin, where 1 means perfect co-localization and 0 no co-localization).

For repair studies, EM9-XH and EM9-V cells were transfected with DNMT1-GFP plasmid, treated with 15 μM 5-azadC for 24 h and then allowed to recover in fresh media for different time points. HeLa cells were similarly transfected, treated and allowed to repair for different time points with or without 1 μM Olaparib. Cells were then fixed using 4% paraformaldehyde and mounted for microscopy. Cells containing more than 10 foci were scored as positive. The data are represented as the percentage of cells with 5-azadC induced foci.

### Ethynyl deoxyuridine (EdU) incorporation to detect replicating cells

HeLa cells were transfected with the *XRCC1*-RFP plasmid and treated with 15 μM 5-azadC for 24 h. In order to quantify the XRCC1 foci-positive cells that were replicating at the end of the treatment, cells were pulse-labeled for 30 min with EdU and then fixed with 4% paraformaldehyde. EdU staining was performed according to manufacturers protocol (CLICK-iT EdU^®^ 488, Invitrogen).

### Resazurin proliferation assay

Resazurin sodium salt (Sigma-Aldrich) was added at a final concentration of 0.1 mg/ml to the cell growth media. The plates were then incubated for 6 h before analysis using fluorescence readout at 535/590 nm in an EnVision Multilabel reader (Perkin Elmer™). The absorbance was normalized against background levels and the data processed in MS Excel by standard techniques before statistical analysis in Compusyn™ to generate combination index plots.

### Flow cytometry

For analysis of cell death by apoptosis, cells were processed according to the instructions in BD FITC Annexin V Apoptosis Detection Kit I (BD Pharmingen™), with the exception of the addition of 7AAD (BD Pharmingen™) instead of propidium iodide to stain DNA. The samples were analyzed in a BD FACS Calibur, detecting Annexin-V in the FL1 and 7AAD in the FL3 channel.

### Cellular fractionation and SDS-PAGE analysis

We performed fractionation as described before (22). To ensure equal loading of chromatin fractioned protein, each time point of the experiment was counted separately using the Bio-Rad TC20™ cell counter. 1 × 10^6^ cells were fractioned for each sample. All protein were separated on sodium dodecylsulphate-polyacrylamide gel electrophoresis (SDS-PAGE) gels and subsequently transferred to nitrocellulose membranes. All subsequent steps were carried out in TBS–Tween (10 mM Tris-HCl, pH 7.6, 150 mM NaCl and 0.05% Tween-20) either containing 5% milk (blocking and antibody incubations) or 5% BSA (phospho-specific antibody incubations). Antibody binding was visualized by enhanced chemiluminescence using the SuperSignal West Dura or Pico reagents from Pierce™ and a Chemidoc MP system from Bio-Rad™. Antibodies used; Actin (Abcam), DNMT1 (Abcam), cleaved-Parp (Cell signaling), γ-H2AX (Millipore), HistoneH3 (Santa Cruz), GAPDH (Abcam) and Ku70 (Santa Cruz).

### *In vitro* 5-azadC-DNA excision assay

Genomic DNA prepared from EM9-V cells exposed to 0.1 μM [6–^3^H]5-azadC was incubated with whole cell extracts prepared by lysing cells on ice in three packed cell volumes in lysis buffer (25 mM Tris-HCl pH 8.0, 200 mM KCl, 2 mM ethylenediaminetetraacetic acid, 1 mM DL-Dithiothreitol (DTT),1× Complete protease inhibitors (Roche), 0.5% v/v NP-40 and 40% glycerol). Following incubation, acid-insoluble material was removed by precipitation in 5% TCA and centrifugation at 16 000 *g* for 15 min. Radioactivity in the supernatant was counted in a Packard Tri-Carb liquid scintillation counter. DNA from cells exposed to [^3^H]TdR was used as control.

### Statistical analysis

To determine the significance of our results, Student's *t*-test was used. Statistical analysis and plotting of the results were performed using GraphPad Plus, Sigma Plot or MS Excel. The results originate from at least two independent experiments and are presented as mean ± standard deviation (SD) of the mean. Differences were considered significant when *P* < 0.05 (*) or *P* < 0.01 (**). Non-statistically significant differences were labeled as n.s.

## RESULTS

### BER deficient cells are hypersensitive to 5-azadC

**Figure 1.**
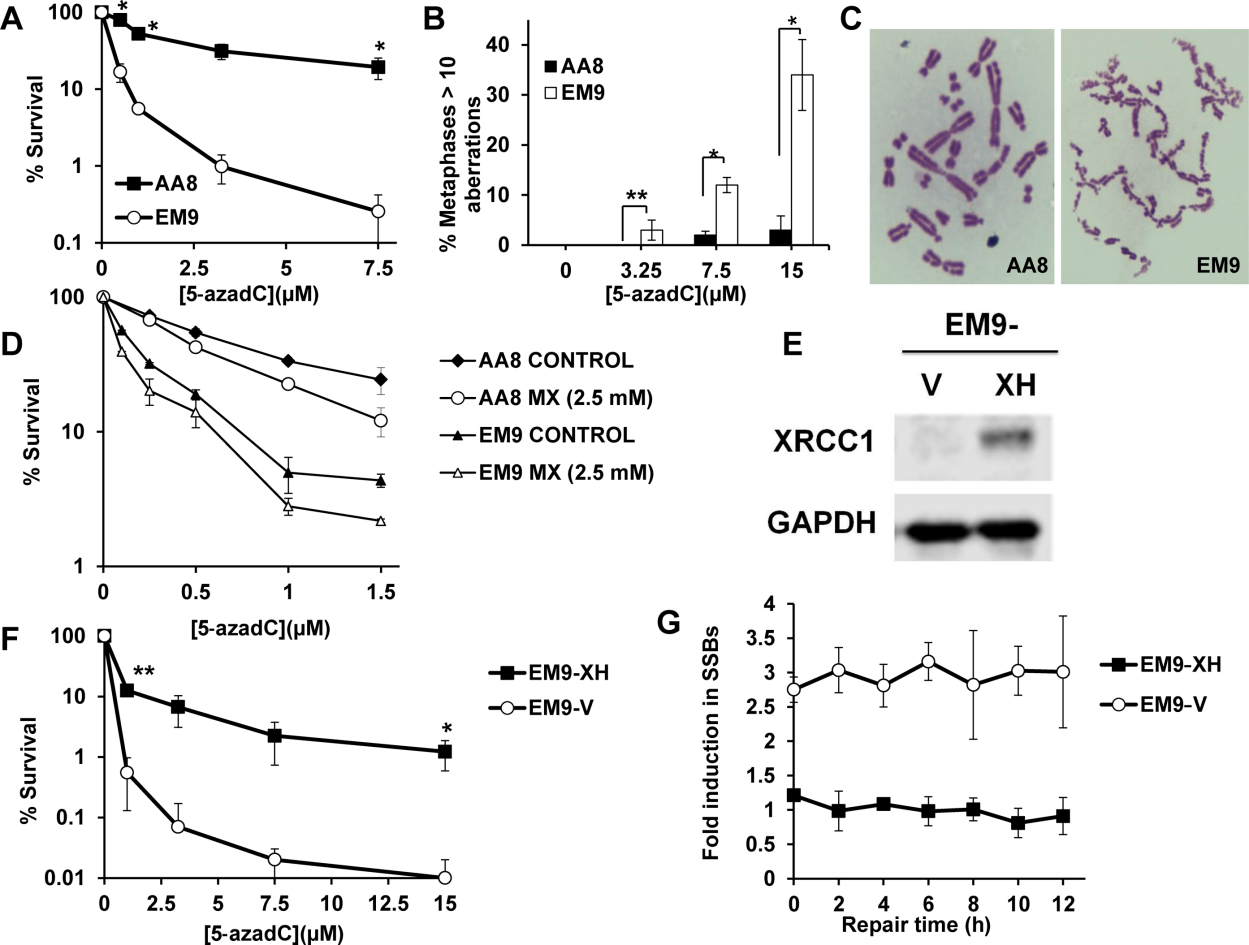
XRCC1 deficient cells are hypersensitive to 5-azadC. (**A**) Clonogenic survival after 24-h treatment with increasing doses of 5-azadC in EM9 (XRCC1 deficient) and AA8 (*XRCC1* wild-type) cells. After treatments, cells were allowed to form colonies in fresh media for 7-days. The means and SD from three independent experiments are shown. (**B**) Quantification of chromosomal aberrations after a 24-h treatment with 5-azadC in AA8 and EM9 cells (wild-type or XRCC1 deficient cells respectively). Cells were treated for 24 h and then allowed to repair for 12 h in drug free media. Cells with 10 or more chromosomal aberrations were scored as positive. The means and SD of two independent experiments are shown. (**C**) Representative metaphases of AA8 and EM9 cells treated with 15 μM 5-azadC. (**D**) MX sensitizes AA8 and EM9 cells to 5-azadC. Cells were treated with increasing concentrations of 5-azadC together with 2.5 mM of MX for 24 h. Media was then changed and cultures were treated again with MX for another 24 h. After 7 days, colonies were fixed and stained using methylene blue. The data are plotted as percentage of survival compared to control cells. The means and SD of two independent experiments are shown. (**E**) XRCC1 detection by western blot in EM9-V and EM9-XH cells. GADPH was used as loading control. (**F**) Clonogenic survival after 24**-**h treatment with increasing doses of 5-azadC in EM9-V (XRCC1 deficient) and EM9-XH (*XRCC1* complemented) cells. After treatments, cells were allowed to form colonies in fresh media for 7 days. The means and SD from two to three independent experiments are shown. (**G**) EM9-XH and EM9-V cells were treated for 24 h with 5-azadC (50 μM) and then allowed to repair in drug-free media for different times at which they were lysed and processed for ADU technique. The data are represented as fold increase in SSBs relative to control cells (value: 1). The means and SD of two independent experiments are shown.

As an aberrant base, it is possible that 5-azadC is recognized and repaired by an excision repair mechanism. There are numerous DNA glycosylases that potentially could exert this function; however, downstream repair depends on a functional XRCC1 protein. To be able to monitor all repair events, we used XRCC1 defective cells to determine a potential role of BER in repair of lesions induced by 5-azadC. Chinese hamster ovary (CHO) EM9 cells deficient for XRCC1 expression and its parental wild-type AA8 cells were plated at low density for clonogenic survival and then treated with increasing doses of 5-azadC. EM9 cells were shown to be significantly more sensitive to 5-azadC than XRCC1 WT AA8 cells (Figure 1A).

We next wanted to determine whether the dramatic sensitization seen in EM9 cells was due to a lack in the repair of 5-azadC induced lesions. Chromosomal aberrations were analyzed in XRCC1 deficient EM9 and parental AA8 cells treated with 5-azadC after a 12-h repair period. Analysis of the percentage of cells with 10 or more aberrations showed a significant difference between AA8 and EM9 cells (Figure 1B and C). At the highest concentration of 5-azadC (15uM), 34% of the EM9 cells and 3% of the AA8 cells displayed more than 10 aberrations (*P < 0.05).

Abasic sites are generated when the BER pathway processes damaged nucleotides. In order to determine if abasic sites are indeed generated, implicating engagement of BER, we probed 5-azadC treated AA8 and EM9 cells with an abasic site specific biotinylated probe (ARP), and detected the fluorescence of biotinylated AP-sites by flow cytometry (23). This analysis pointed to an increase in abasic sites independent of XRCC1 status (Supplementary Figure S1). As the ARP probe also presents reactivity with carbonyl groups present in proteins, we analyzed the ARP fluorescence by microscope and found that 5-azadC treated cells present more fluorescence in the nucleus as compared to control cells, which suggest that abasic sites are generated (Supplementary Figure S2). To further show that abasic sites are generated, we utilized the abasic site specificity of the drug MX (24). MX creates adducts by binding to the abasic site and inhibiting the subsequent incision by APE1. If the lesions induced by 5-azadC are repaired through the BER pathway, sub-lethal doses of MX would cause synergistic lethality in combination with 5-azadC (25). Indeed, we observed a sensitization to 5-azadC in XRCC1 WT AA8 cells and EM9 cells after a sub-lethal dose of MX treatment pointing to an involvement of the BER pathway (Figure 1D and Supplementary Figure S3A). This effect was also dose dependent as increasing the dose of MX further sensitized AA8 cells to 5-azadC (Supplementary Figure S3B).

We next wanted to validate our data with EM9 cells complemented with XRCC1 WT expression. EM9 cells either stably transfected with either an empty vector (EM9-V) or with a vector containing WT human *XRCC1* (EM9-XH) were treated with 5-azadC and plated for clonogenic survival. Indeed, EM9-V cells were found to be more sensitive to treatment than XRCC1 complemented EM9-XH cells (Figure 1E and F). These cells were also found to be hypersensitive to 5-azaC, another synthetic cytidine analog (Supplementary Figure S4).

Because XRCC1 deficient cells are defective in the repair of SSBs (13,14) we also wanted to examine the repair kinetics of SSBs using the alkaline DNA unwinding (ADU) technique (15,19). XRCC1 deficient cells treated with 5-azadC displayed increased levels of strand breaks that persisted for 12 h post treatment compared to complemented cells, in which we could not find a substantial induction in SSBs (Figure 1G).

In conclusion, our data suggest that BER is an active mechanism in the repair of 5-azadC lesions.

### BER deficiency leads to induction of DSBs that are substrates for homologous recombination after 5-azadC treatment

**Figure 2.**
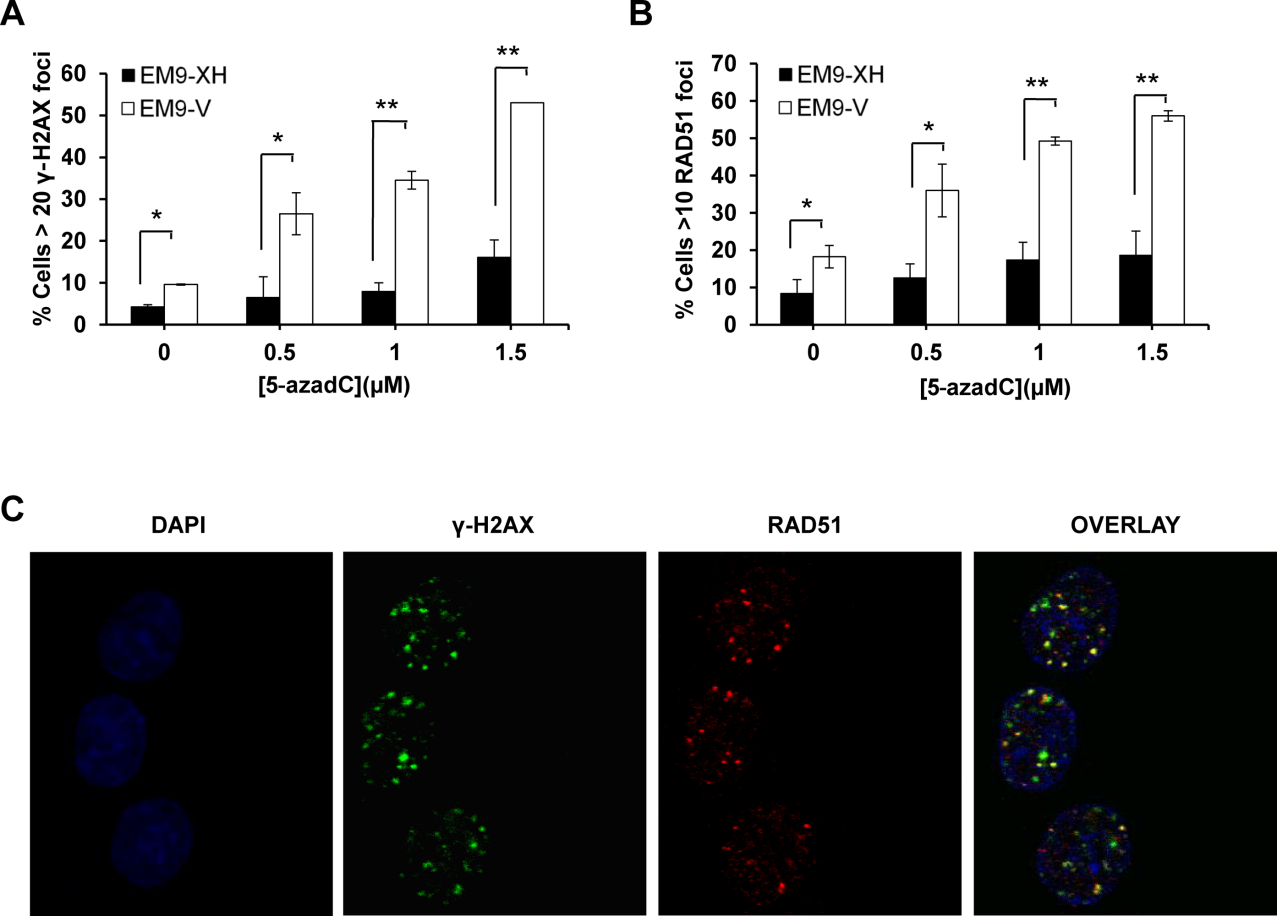
XRCC1 deficient cells display increased levels of γ-H2AX and RAD51 foci induced by 5-azadC. (**A** and **B**) Quantification of γ-H2AX and RAD51 foci positive cell in EM9-XH and EM9-V cells after a 24**-**h treatment with increasing concentrations of 5-azadC. Cells with more than 20 or 10 foci respectively were scored as positive respectively. The means and SD of three independent experiments are shown. (**C**) Co-localization between γ-H2AX and RAD51 foci induced by 5-azadC.

When a replication fork encounters a SSB or an unrepaired base lesion, one potential outcome collapses and it converts into a DSB (12). These collapsed forks are known substrates for homologous recombination (HR) (26). BER (XRCC1) deficient cells have been reported to have increased spontaneous levels of γ-H2AX and RAD51 foci, which are markers of DSBs and HR respectively (12). We could observe increased levels of both γ-H2AX and RAD51 foci at all concentrations of 5-azadC tested in XRCC1 deficient cells (EM9-V) (Figure 2A and B, *P* < 0.05 and *P* < 0.01). γ-H2AX and RAD51 foci co-localized (Manders co-localization index = 0.84 ± 0.09, *n* = 20), showing that the induced DSBs are indeed repaired by HR (Figure 2C). Taken together, these data demonstrate that in the absence of XRCC1, 5-azadC induced lesions result in higher levels of DSBs and HR repair activation.

### XRCC1 co-localizes with trapped DNMT1 after 5-azadC treatment

**Figure 3.**
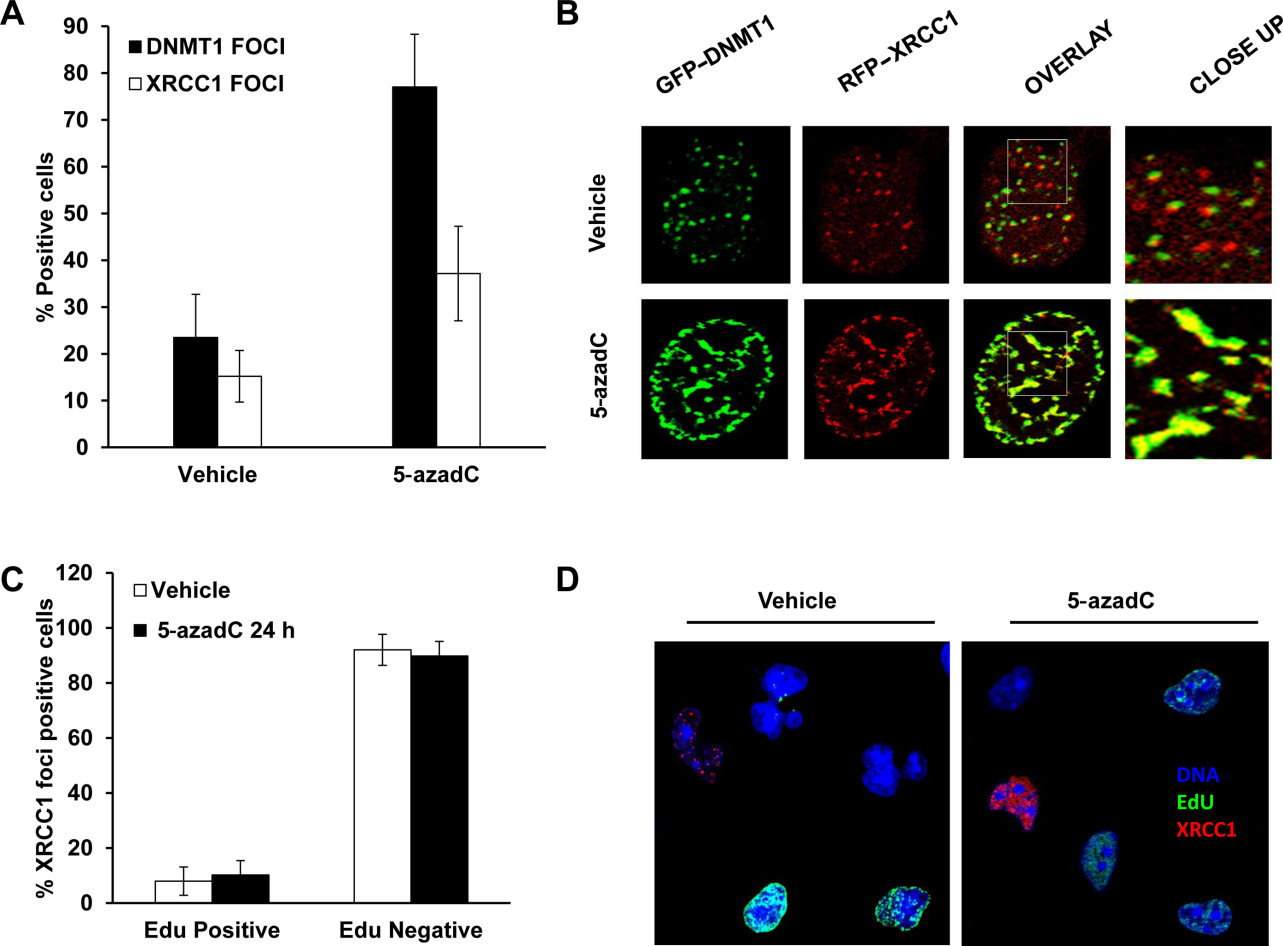
XRCC1 co-localizes with DNMT1 foci preferentially in non-replicating cells. (**A**) Quantification of cells positive for DNMT1 or XRCC1 foci after treatment with 5-azadC. Cells with more than 10 foci were scored as positive. The means and SD of three independent experiments are shown. (**B**) Co-localization of DNMT1 and XRCC1 foci in control and 5-azadC treated cells assessed by confocal microscopy. HeLa cells were transiently transfected with plasmids encoding for DNMT1-GFP or XRCC1-RFP and treated with 5-azadC for 24 h. Overlaid images showing yellow signal are indicative of co-localization between green and red foci. Experiments were repeated three times and representative images are shown. (**C**) In order to test if 5-azadC induced XRCC1 foci are associated with replicating cells, XRCC1-RFP expressing cells were treated with 5-azadC for 24 h and incubated with EdU during the last 30 min in order to label replicating cells (green signal). Slides were counterstained with DAPI (blue). The percentage of positive cells for XRCC1 foci (red) that were replicating at the end of the treatment are represented in (C). Similary to control cells, most of the positive cells for XRCC1 foci are not replicating. Representative images are shown in (D).

It is well known that DNA damage caused by 5-azadC is mediated by the trapping of DNMTs and subsequent formation of protein–DNA adducts (6,8). Considering the dramatic phenotype observed when treating XRCC1 deficient cells with 5-azadC, we wanted to test if XRCC1 is directly involved in this repair. We transfected HeLa cells with two separate plasmids expressing GFP-tagged DNMT1 and RFP-tagged XRCC1 and treated them with 5-azadC for 24 h. As previously reported (6), 5-azadC treatment induced a dramatic accumulation of DNMT1 foci (Figure 3A). We also observed an increase in XRCC1 foci, which is in agreement with the responsiveness of cells to 5-azadC. Interestingly, our data also revealed a high level of co-localization of XRCC1 and trapped DNMT, suggesting that XRCC1 plays a role in the repair of 5-azadC induced DNMT1 adducts (Figure 3B). Since both XRCC1 and DNMT1 are proteins known to be associated with active replication forks we wanted to exclude the possibility that the observed interaction occurred at active replication forks using the thymidine analog EdU (27,28). Indeed, the interaction between XRCC1 and DNMT1 following 5-azadC treatment was shown to be independent of DNA replication, since XRCC1 positive cells did not stain positive for EdU (Figure 3C and D). In conclusion, our data suggest that BER proteins have an active role in the repair of 5-azadC trapped DNMT1–DNA adducts.

### Repair of DNMT1–DNA adducts is delayed in an XRCC1 dependent manner

**Figure 4.**
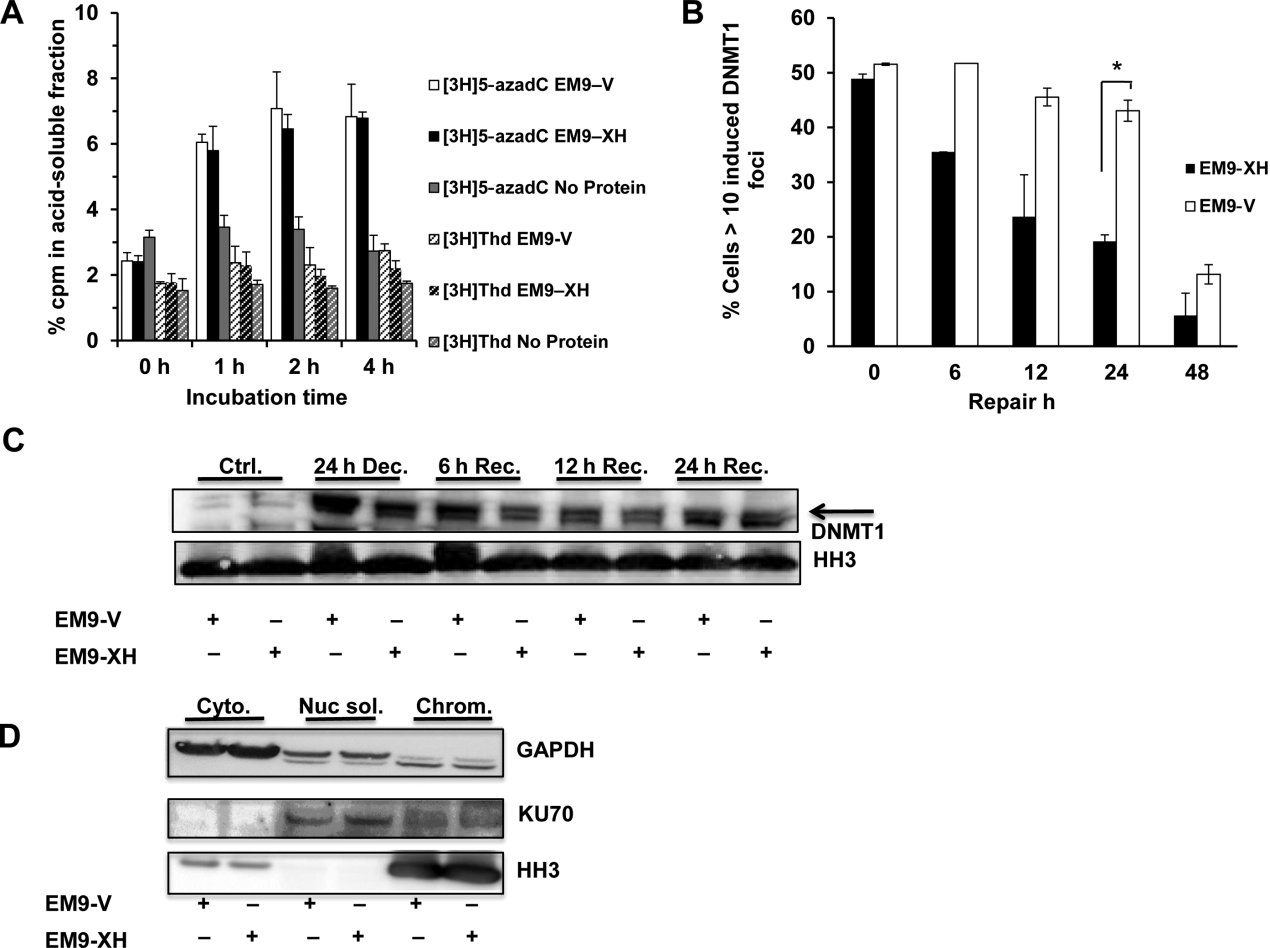
5-azadC cleaved from DNA and 5-azadC induced DNMT1 foci accumulate in XRCC1 defective cells. (**A**) EM9-V cells were treated with (6H-^3^H) 5-azadC and genomic DNA extracted. A known amount of genomic DNA was then incubated with whole cell lysates from EM9-V or EM9-XH cells for the indicated time points. Insoluble DNA was precipitated and the resulting supernatant was analyzed for presence of 5-azadC in a liquid scintillation counter. The standard and means of three independent experiments are shown. (**B**) In order to test if XRCC1 could be related with the repair of trapped DNMT1, EM9-XH and EM9-V cells were transiently transfected with DNMT1-GFP plasmid and then treated with 15 μM of 5-azadC for 24 h. After that, they were allowed to recover for different times at which they were fixed and scored for DNMT1 foci. Cells with more than 10 foci were scored as positive. The data represent the percentage of induced positive cells compared to control cells. The data from two independent experiments are shown. (**C**) XRCC1 deficient cells show a delay in the repair of chromatin bound DNMT1. Exponentially growing EM9-XH and EM9-V cells were treated for 24 h with 15 μM of 5-azadC, washed and allowed to repair for different times at which they were lysed and processed for subcellular fractionation. (**D**) Validation of the fractionation protocol showing the expected localization of GADPH, Ku70 and Histone H3 in the cytosolic- (cyt) nuclear soluble-, (nuc.sol.) and chromatin- (chrom.) enriched fractions, respectively.

We propose that BER has a role in the repair of incorporated 5-azadC; if this is indeed the case, one would expect 5-azadC to be excised from DNA following repair. To monitor this, we incubated [6–^3^H] 5-azadC treated EM9-V genomic DNA with EM9-V and -XH whole cell lysate and then precipitated insoluble DNA and analyzed radioactivity in the remaining supernatant. Both EM9 V and -XH lysate were able to release radioactivity from DNA containing [6–^3^H] 5-azadC but not [^3^H]TdR, demonstrating that excision of 5-azadC precedes the generation of abasic sites in a XRCC1 independent manner (Figure 4A). We next wanted to see if DNMT1–DNA adducts persist for longer times in XRCC1 deficient cells and if this would help explain the observed increase in DSBs. We monitored the repair of DNMT1 foci in EM9-V and -XH cells transfected with a DNMT1-GFP construct. The cells were treated for 24 h with 5-azadC and then left to recover in fresh media (Figure 4B). Both cell lines displayed similar levels of DNMT1 foci positive cells after the treatment with 5-azadC. However, EM9-XH repaired the DNMT1 foci faster than the XRCC1 deficient EM9-V cells over the course of 48 h, indicative of defective repair in the XRCC1 deficient setting. To verify these findings, we also performed a chromatin fractionation assay to determine the amount of endogenously expressed DNMT1 bound to chromatin after 5-azadC treatment. XRCC1 deficient EM9-V cells displayed more chromatin-bound DNMT1 with delayed repair compared to complemented EM9-XH cells (Figure 4C and D and Supplementary Figure S5A). When looking at EM9-V and -XH cells transfected with DNMT1-GFP, similar levels of chromatin bound DNMT1 were found after 5-azadC exposure but again, EM9-V cells displayed slower repair kinetics (Supplementary Figure S5B). In conclusion, our data demonstrate that BER is activated following 5-azadC exposure and that the repair kinetics of chromatin bound DNMT1 and randomly incorporated 5-azadC is severely affected in XRCC1 deficient cells, leading to an increase in DSBs.

### PARP inhibition abolishes 5-azadC-induced XRCC1/DNMT1 co-localization, interrupts BER and generates DSBs which are repaired by HR.

**Figure 5.**
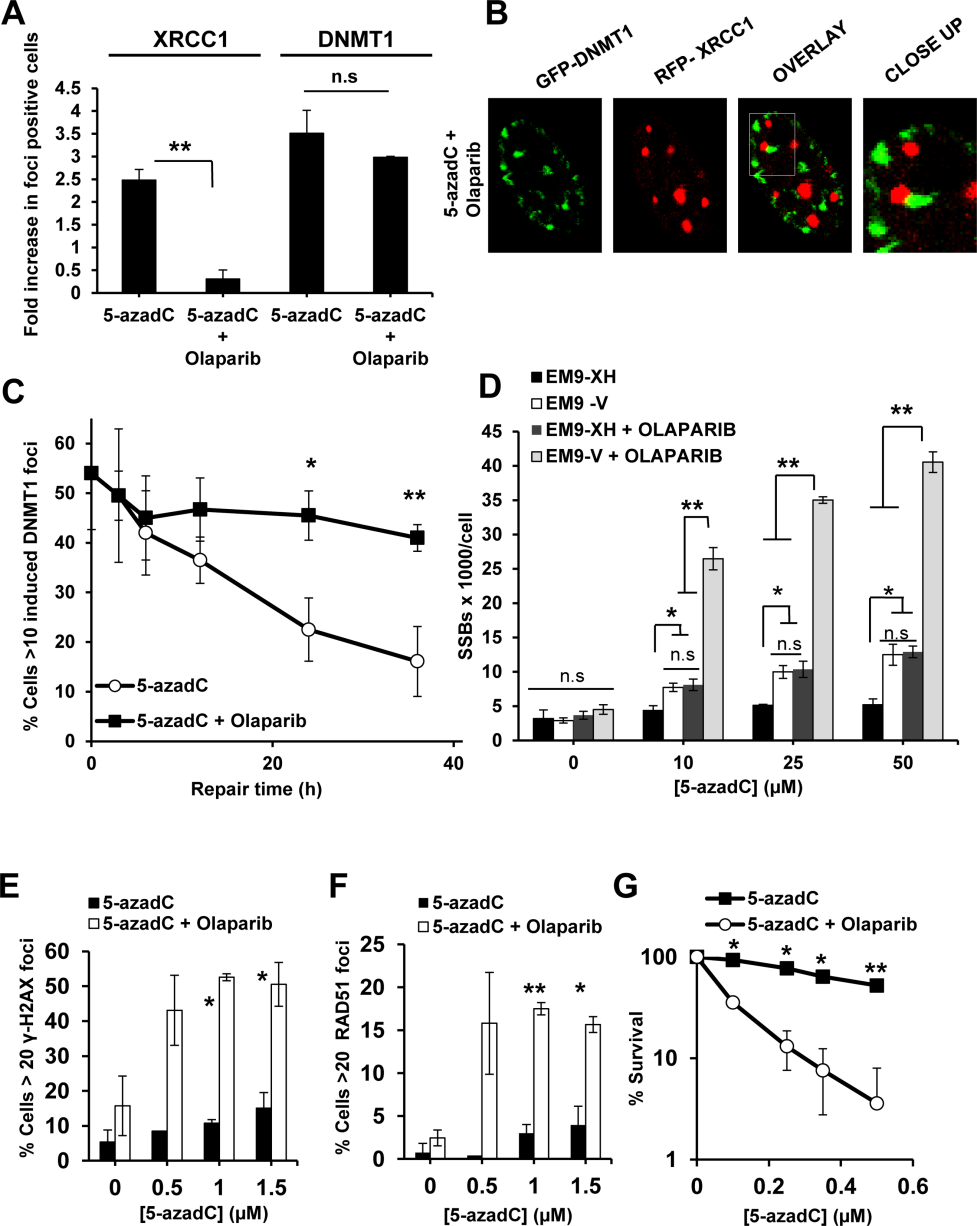
Olaparib disrupts DNMT1/XRCC1 co-localization and traps SSBs intermediates of BER in cells treated with 5-azadC. (**A**) Quantification of cells positive for DNMT1 or XRCC1 foci after treatment with 5-azadC either alone or in combination with Olaparib. Cells with more than 10 foci were scored as positive. The data are represented as fold increase in positive cells relative to control cells (value: 1). The means and SD of three independent experiments are shown. (**B**) Co-localization of DNMT1 and XRCC1 foci in 5-azadC cells co treated with Olaparib as assessed by confocal microscopy. HeLa cells were transiently transfected with plasmids encoding for DNMT1-GFP or XRCC1-RFP and treated with 5-azadC in combination with Olaparib for 24 h. No sign of co-localization was observed in overlaid images. Experiments were repeated three times and representative images are shown. (**C**) HeLa cells expressing the DNMT1-GFP plasmid were treated with 15 μM of 5-azadC for 24 h. After that, they were able to recover in fresh media with or without the presence of 1 μM of Olaparib for different times at which they were fixed and scored for DNMT foci. Cells with more than 10 foci were scored as positive. The data represent the percentage of induced positive cells compared control cells. The data from two independent experiments are shown. (**D**) SSB quantification using ADU technique after a 24-h treatment with increasing concentrations of 5-azadC. EM9-XH and EM9-V cells were treated with 5-azadC alone or in combination with of the PARP inhibitor Olaparib (1 μM) during the last 12 h. The means and SD of three independent experiments are shown. (**E** and **F**) Quantification of γ-H2AX and RAD51 foci positive cells after a 24-h treatment with increasing concentrations of 5-azadC alone or in combination with Olaparib (1μM). Cells with more than 20 foci were scored as positive. The means and standard errors of two independent experiments are shown. (**G**) Clonogenic survival of HeLa cells with increasing concentrations of 5-azadC for 24 h alone or in combination with Olaparib (0.3 μM). After that cells were washed and treated again with Olaparib until the fixation day. The means and standard deviation of two independent experiments are shown. Statistical significance was determined using Student's *t*-test, n.s. = non-significant, **P < 0.01, *P < 0.05.

We and others have previously shown that PARP-1 has the capacity to bind the SSB intermediates formed during BER (15,16). PARP inhibition traps PARP-1 at the SSB intermediate, thus creating an efficient block of further repair (15,16,29). PARP inhibitors are currently being evaluated in clinical trials because of the synthetic lethality caused in cancers with genetic makeups that renders HR repair inactive, such as BRCA1 or BRCA2 mutations (30,31). Due to SSB repair defects in PARP inhibited cells and the obvious clinical interest in PARP inhibitors, we wanted to evaluate if PARP inhibition could block 5-azadC-induced BER and sensitize cells to treatment. To this end, we transfected HeLa cells with plasmids expressing GFP-tagged DNMT1 and RFP-tagged XRCC1. Co-treatment with 5-azadC and Olaparib led to a dramatic reduction in the ratio of XRCC1 but not DNMT1 foci compared to 5-azadC treated cells (Figure 5A), in agreement with the proposed function of PARP in the assembly of XRCC1 (32). Furthermore, when we first treated these cells with Olaparib, we found no co-localization between XRCC1 and DNMT1, suggesting a role of PARP in the loading of XRCC1 onto trapped DNMT1 (compare Figure 5B and Figure 3B, Supplementary Figure S6). These data suggest that PARP inhibition disrupt the repair of 5-azadC induced lesions. We next wanted to know if the repair of 5-azadC induced DNMT1–DNA adducts is affected by PARP inhibition. Clearly, PARP inhibited cells displayed a severe defect in the clearance of DNMT1 foci (Figure 5C). Also, SSBs induced by 5-azadC in EM9-XH cells (*XRCC1* wild-type) were increased in the presence of Olaparib (Figure 5D). We also observed that EM9-V and Olaparib treated EM9-XH cells displayed similar levels of SSBs, indicating an equally important role for these proteins in repair of 5-azadC induced lesions. Moreover, when cells were treated with 10 μM of 5-azadC together with Olaparib, we observed a synergistic accumulation of SSBs in XRCC1 deficient cells (average 27 000 versus 9000 SSBs respectively, *P* < 0.01) (Figure 5D). This data agrees with our previous hypothesis that PARP inhibitors trap SSB intermediates of BER (15). Similar data were obtained using 4-ANI, another PARP inhibitor (Supplementary Figure S7). Importantly, SSBs were detected 2 h after 5-azadC exposure in 4-ANI treated cells, showing that SSBs are early intermediates of repair (Supplementary Figure S8).

Next, we wanted to investigate if trapped intermediates of BER could be converted into DSBs and trigger HR. In HeLa cells, a combined treatment of 5-azadC and Olaparib lead to increased levels of γ-H2AX and RAD51 foci (Figure 5E and F) as compared with single treatments, suggesting that HR has a role in the repair of 5-azadC lesions when PARP is inhibited. This increase in DNA damage also dramatically reduced cell survival (Figure 5G). In conclusion, our data show that PARP inhibition severely disrupts the BER pathway following 5-azadC and that this damage engage HR.

### PARP inhibition in combination with 5-azadC cause synthetic lethality in acute myeloid leukemia cells

**Figure 6.**
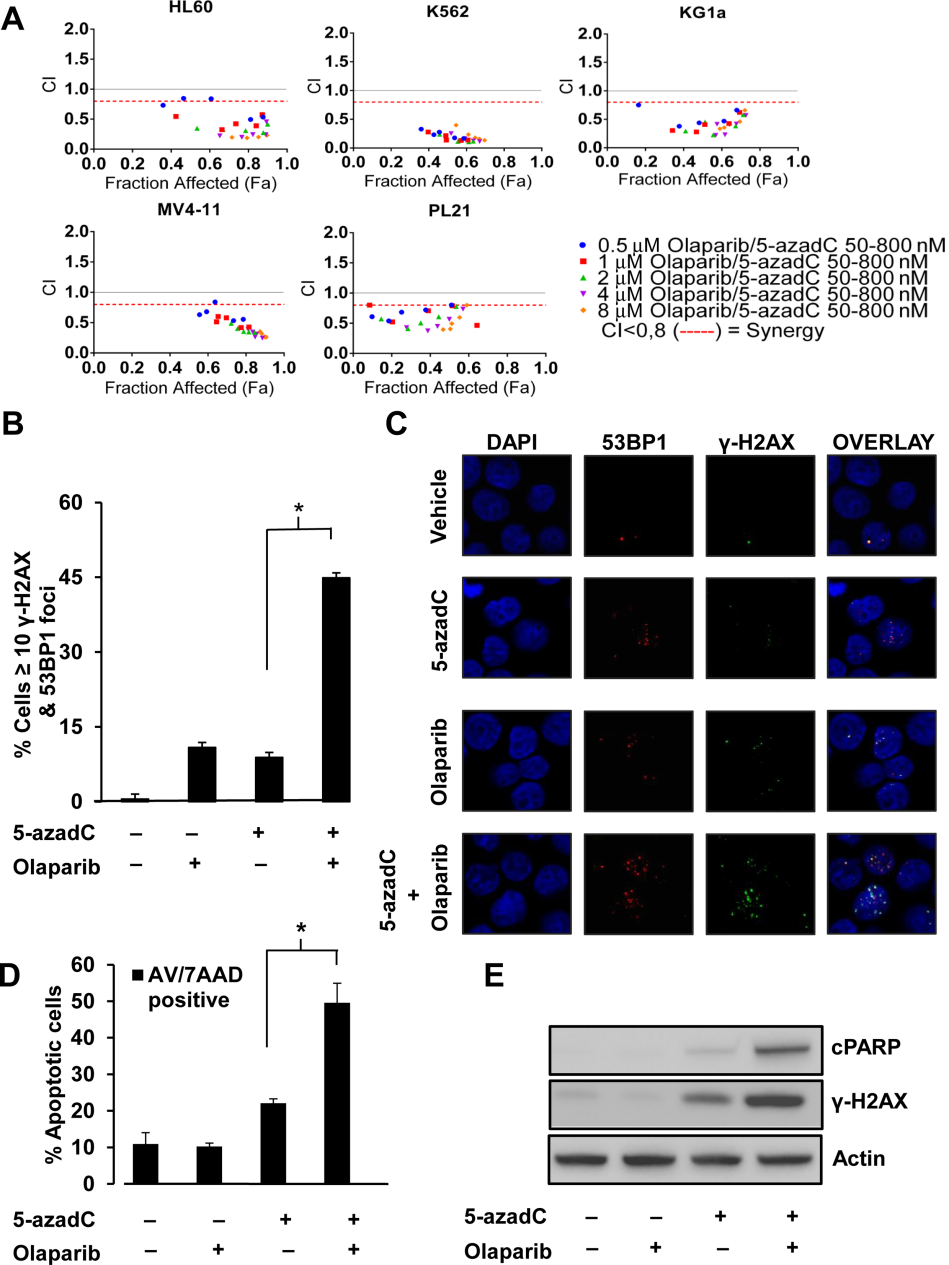
5-azadC and Olaparib cause synergistic lethality in a panel of AML cell lines. (**A**)The AML cell lines HL60, K562, Mv4-11, KG1a and PL21 were incubated at optimal growth conditions in 96-well plates with either 5-azadC (50, 100, 200, 400 and 800 nM) or Olaparib (0.5, 1, 2, 4 and 8 μM) or in combination at all concentrations. After 72 h in the presence of the treatment, Resazurin was added and the absorbance measured. The Combination Index (CI) was plotted against the Fraction affected (Fa) using the software Compusyn™, where CI < 0.8 is considered synergistic lethal (Red dotted line). (**B**) Quantification of γ-H2AX and 53Bp1 foci positive cells after a 24 h treatment with 100nM of 5-azadC alone or in combination with Olaparib (1 μM). The means and SD of two independent experiments are shown. (**C**) Representative images of γ-H2AX and 53Bp1 foci in HL60 cells. (**D**) The AML cell line HL60 was incubated at optimal growth conditions with a combination of 250 nM 5-azadC and 2 μM Olaparib for 48 h, single treatments of 5-azadC and Olaparib or the combination of both was quantified for both early (Annexin-V) and late apoptotic cells (Annexin-V + 7AAD). The data show a significant increase in the induction of apoptotic cells in the combination group. The means and SD of three independent experiments are shown. (**E**) Cells were also harvested for SDS-PAGE analysis. The combination treatment shows increased DNA damage (γ-H2AX) and induction of apoptosis (cleaved-PARP). *P < 0.05.

5-azadC (Decitabine) is an FDA approved drug for the treatment of MDS and AML (33). The use of this demethylating agent has proved to be a significant improvement over previous best supportive care (34), but there are still non-responding patients and those developing resistance to therapy. In light of this, we wanted to evaluate if combining Olaparib with 5-azadC in established human AML cell lines would render 5-azadC treatments more efficient. We used a human AML cell line panel consisting of HL60, k562, MV4–11, PL21 and KG1a. The cells were treated for 72 h, after which the synthetic lethality of the drug–drug combination was measured by the addition of resazurin and analyzed using the drug combination algorithm by Chou and Talalay (35) (Figure 6A). Although some datapoints scored as additive (primarily in PL21 cells), a majority of the datapoints in the other cell lines scored as synthetic lethal (CI < 0.8, red line). We next wanted to investigate if the 5-azadC and Olaparib combination induced double stranded DNA damage, similarly to what we observed in HeLa cells. The HL60 cell line was treated with Olaparib and 5-azadC alone or in combination and then scored for co-localization of the double stranded DNA damage markers γ-H2AX and 53Bp1. The combination treatment displayed a synergistic induction of double stranded DNA damage (Figure 6B and C, *P* < 0.05). We also stained the cells for the early apoptotic marker phosphatidylserine using Annexin V in combination with 7-AAD after 48 h exposure to the drug combination. Quantification of the double positive cell population (7AAD/Annexin V) in the treatment groups showed a significant increase in the 5-azadC and Olaparib group as compared to the single drug treated cells (Figure 6D, *P* < 0.05), with an increase of cleaved PARP and phosphorylation of H2AX also evident (Figure 6E). The cells also displayed a progressive presentation of phosphatidylserine on the outer membrane with a later uptake of the DNA dye 7AAD, proving that the cells die by apoptosis (Supplementary Figure S9).

## DISCUSSION

There are different non-exclusive cytotoxic mechanisms of action of 5-azadC; epigenetic changes in gene expression causing cell death, trapping of DNMT onto DNA causing lethal DNA damage and DNA damage generated by randomly incorporated 5-azadC. Here, we wanted to determine if BER could play a role in repair of 5-azadC-induced DNA lesions and also if it could promote survival. We utilized the CHO BER (XRCC1) deficient cell system and demonstrate the novel finding that XRCC1 deficiency sensitizes cell to 5-azadC treatment. We also demonstrate that blocking BER with the PARP inhibitor Olaparib cause synergistic lethality in HeLa cells and a panel of acute myeloid leukemia cell lines.

**Figure 7.**
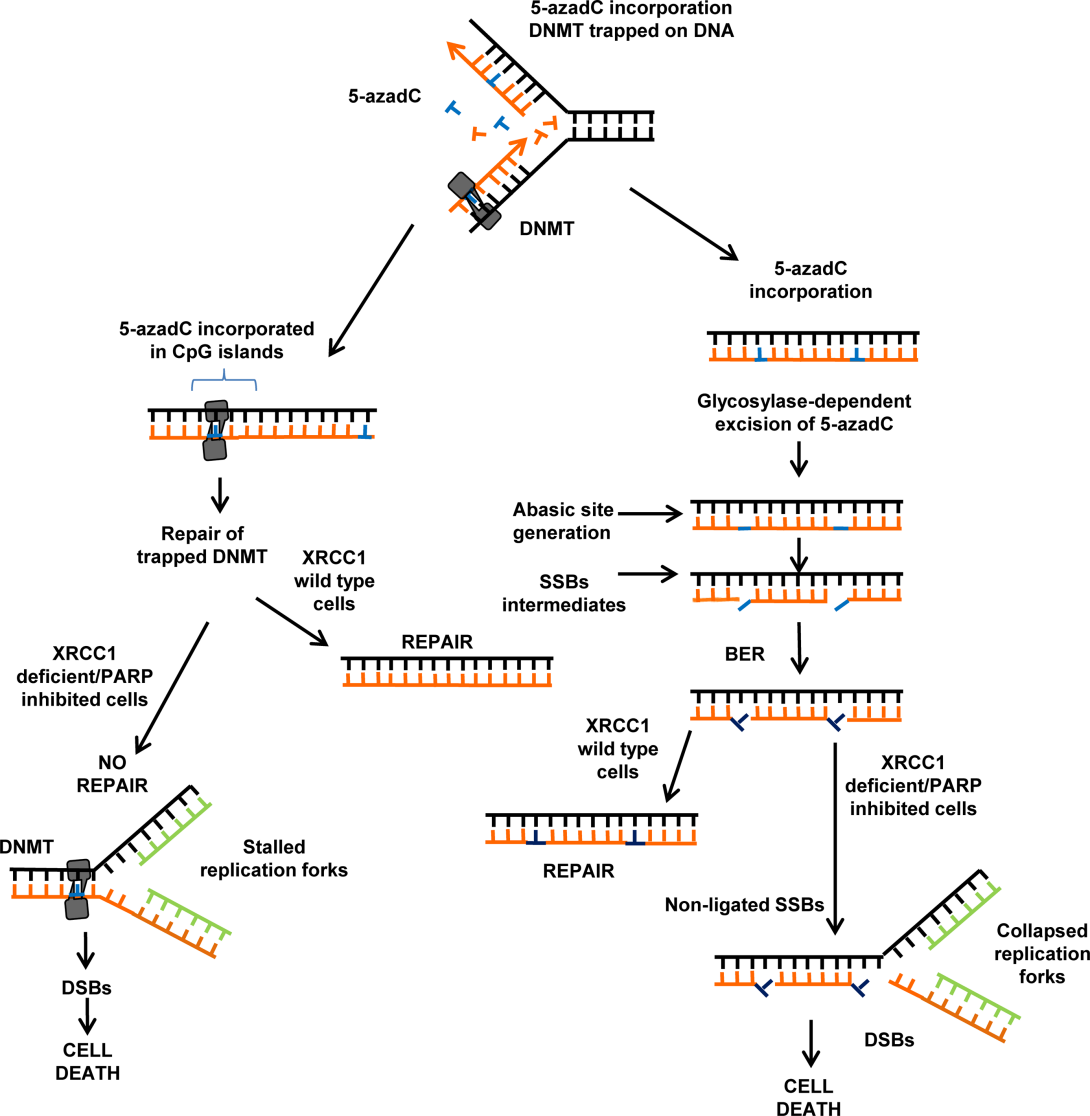
Proposed model involving XRCC1 in the repair of 5-azadC induced lesions. 5-azadC is incorporated in CpG islands and randomly in the newly formed daughter strand during replication. 5-azadC is excised from DNA independently of XRCC1 status, but in conditions of defective XRCC1 or PARP inhibited cells, XRCC1 fails to localize to DNMT1 bound DNA, delaying repair. In XRCC1 deficient or PARP inhibited cells, delayed repair and non-sealed ssDNA following 5-azadC excisions leads to replication fork collapse, DSBs and cell death.

A model for our proposed mechanism of action is presented in Figure 7. 5-azadC is incorporated in CpG islands during normal replication as well as randomly in the newly formed daughter strand instead of normal cytosine. Our data demonstrate that both the excision of 5-azadC from DNA and the generation of abasic sites occur independently of XRCC1 status. However, XRCC1 deficient cells display increased levels of SSBs and show a synergistic increase in SSBs when PARP inhibitors are present. Taken together, these data demonstrate that BER is actively removing 5-azadC from DNA. However, we believe that DNMT1–DNA adducts are most likely too big to be removed by DNA glycosylases and are perhaps removed by a base damage repairing enzyme like Tyrosyl DNA Phosphodiesterase 1 (TDP1) (36) or TDP2 instead (37). Our data also show that DNMT1 foci co-localize with XRCC1. This could point to a previously unidentified role of XRCC1 in the clearance of bound DNMT1 from DNA. We tested this possibility by two different approaches and found that XRCC1 deficient cells have delayed clearance of DNMT1–DNA adducts. This body of data, although robust, is not enough to directly implicate XRCC1 in the repair of DNMT1–DNA adducts. Theoretically, after 5-azadC treatment, CpG islands could present clustered DNA damage comprised of DNMT adducts and SSBs generated form excision of naked 5-azadCTP. These SSBs could potentially hinder the repair of trapped DNMT especially when accumulated, as is the case in XRCC1 deficient cells.

Because PARP activity is required for efficient relocation of XRCC1 to damaged sites (32), the XRCC1-DNMT1 co-localization is abolished in the presence of PARP inhibitors, causing delay in repair, increased DNA damage and synthetic lethality in combination with 5-azadC.

Many patients receiving 5-azadC relapse or develop resistance. Therefore, we reason that any combination that increases the effect of this hypomethylating agent should be carefully evaluated as a potentially improved therapeutic opportunity. In recent years, PARP inhibitors have become one of the most interesting options in the treatment of *BRCA*-mutant cancers (38,39). The mechanism of action implicates a novel mechanism involving DNA repair. In clinical trials, PARP inhibitors are used either as monotherapy for the treatment of *BRCA-*mutated cancers or in combination with other chemotherapeutics for the treatment of solid cancers such as pancreatic, colorectal and cervical cancer (39). Here, we show that PARP inhibition using Olaparib perturbs BER in a XRCC1 dependent manner and causes synergistic lethality with 5-azadC in acute myeloid leukemia cell lines. Others have previously shown modest effect of this combination treatment in some of the same model systems using other PARP inhibitors (40). We believe that the additive effect demonstrated in that report is largely due to dosing, as we also observe an additive effect at lower doses of Olaparib. Nevertheless, our data clearly suggest that perturbation of BER using Olaparib could be a potential approach to increase efficacy of 5-azadC treatment. Current applied maximum tolerated doses of 5-azadC have been established based on the wide range of side effect it exerts on disease free proliferating tissue. We think that the full therapeutic potential of 5-azadC and related agents have yet to be discovered because of this. By targeting therapy to stratified patients carrying XRCC1 deficiency, or combining 5-azadC with a PARP inhibitor we could improve the effects of the hypomethylating agent.

An increasing number of reports have shown a correlation between single nucleotide polymorphism (SNP) of the *XRCC1* gene and the risk to develop various cancers, including AML (41,42). There is evidence that some of the *XRCC1* polymorphisms found in cancer cells correlate with defects in the repair of DNA damage induced by several anti-cancer agents (43–45). Our data clearly indicate that XRCC1 deficiency perturbs the BER pathway and sensitizes cancer cells to 5-azadC treatment. It would be interesting to investigate if *XRCC1* polymorphisms correlate with hypersensitivity to 5-azadC. If this is the case, AML patients carrying defects in XRCC1 function could be stratified for 5-azadC treatment, resulting in a more effective option.

Taken together, our results offer evidence for an important therapeutic potential of BER deficiency and PARP inhibition when combined with 5-azadC. Our data warrant further investigation in order to improve the already positive trends seen when treating myeloid malignancies with 5-azadC and 5-azaC.

## SUPPLEMENTARY DATA

Supplementary Data are available at NAR Online.

SUPPLEMENTARY DATA
