# Supplementary material for: The PARP inhibitor Olaparib disrupts base excision repair of 5-aza-2′-deoxycytidine lesions
Source: Nucleic Acids Res. 2014 Jul 29;42(14):9108–20. doi: 10.1093/nar/gku638 (PMC4132747; doi:10.1093/nar/gku638)
Supplement: SUPPLEMENTARY DATA [file supp_42_14_9108__index.html]

The PARP inhibitor Olaparib disrupts base excision repair of 5-aza-2′-deoxycytidine lesions — SUPPLEMENTARY DATA 

# The PARP inhibitor Olaparib disrupts base excision repair of 5-aza-2′-deoxycytidine lesions

## SUPPLEMENTARY DATA

**Files in this Data Supplement:**

- SUPPLEMENTARY DATA
