# Supplementary material for: The PARP inhibitor Olaparib disrupts base excision repair of 5-aza-2′-deoxycytidine lesions
Source: Nucleic Acids Res. 2014 Jul 29;42(14):9108–20. doi: 10.1093/nar/gku638 (PMC4132747; doi:10.1093/nar/gku638)

SUPPLEMENTARY INFORMATION

THE PARP INHIBITOR OLAPARIB DISRUPTS BASE EXCISION REPAIR OF 5-AZA-2´-DEOXYCYTIDINE LESIONS.

Manuel Luis Orta[1,2]†, Andreas Höglund[2]†, José Manuel Calderón-Montaño[2,3], Inmaculada Domínguez[1], Estefanía Burgos-Morón[3], Torkild Visnes[2], Nuria Pastor[1], Cecilia Ström[2], Miguel López-lázaro[3] and Thomas Helleday[2]*]

[1] *Department of Cell Biology, Faculty of Biology, University of Seville, Avda. Reina Mercedes 6, 41012, Seville, Spain.*

[2] *Science for Life Laboratory, Division of Translational Medicine and Chemical Biology, Department of Medical Biochemistry and Biophysics, Karolinska Institute, S-171 21 Stockholm, Sweden.*

[3] *Department of Pharmacology, Faculty of Pharmacy, University of Seville, c/ Professor García González, nº 2, 41012, Seville, Spain.*

† These authors contributed equally to this work

* To whom correspondence should be addressed. Professor Thomas Helleday, PhD. Torsten and Ragnar Söderberg Professor of Translational Medicine. Office: +46 8 524 80000, E-mail: thomas.helleday@scilifelab.se. Science for Life Laboratory, Division of Translational Medicine and Chemical Biology. Department of Medical Biochemistry and Biophysics, Karolinska Institutet, Box 1031, S-171 21 Stockholm, Sweden.

SUPPLEMENTARY FIGURE LEGENDS:

SUPPLEMENTARY Figure 1. Flow cytometric measurement of the relative amount of abasic sites in wild type AA8 cells and XRCC1 deficient cells. Cells were treated with 5-azadC for 24 hours and processed according to the AP site detection kit (Abcam). FITC fluorescence was determined using a Beckman Coulter cytomics FC 500 MPL flow cytometer (FL1 channel).

SUPPLEMENTARY Figure 2. Incidence of abasic sites in AA8 and EM9 cells treated with 50 µM of 5-azadC for 24 hours. As can be seen, 5-azadC treated cells present more fluorescence in the nucleus as compared with control cells which points to the generation of abasí sites in both cell lines.

SUPPLEMENTARY Figure 3. MX sensitizes cells to 5-azadC. (A) Plating efficiency of AA8 cells treated with MX for 48 hours (media was changed and new MX was added at 24 hours). (B) Clonogenic survival of AA8 cells treated with increasing concentrations of 5-azadC together with MX for 48 hours (replenishment after 24 hours). After 7 days, colonies were fixed and stained using methylene blue. The data are plotted as percentage of survival compared to control cells. The means and SD of three independent experiments are shown.

SUPPLEMENTARY Figure 4. XRCC1 deficient cells are hypersensitive to 5-azaC. Clonogenic survival after 24 hour treatment with increasing doses of 5-azaC in EM9-V (XRCC1 deficient) and EM9-XH (XRCC1 complemented). After treatment, cells were allowed to form colonies in fresh media for 7 days. The means and SD from one to two independent experiments are shown.

SUPPLEMENTARY Figure 5. (A) Quantification of WB presented in Figure 4C. After 5AzadC treatment, EM9V cells show more DNMT1 bound to chromatin with slower repair kinetics compared to EM9XH cells. Data is normalized against control and presented in arbitrary units (AU). (B) Slower repair of DNMT1-DNA adducts in XRCC1 deficent cells. EM9-V (XRCC1 deficient) and EM9-XH (*XRCC1* complemented) cells were transfected with a plasmid expressing DNMT-GFP. After 24h exposure to 5-azadC, the cells were allowed to recover for the indicated time points. Quantification of DNMT1 bound to DNA after 24 hours 5-azadC treatment show little

difference between EM9-V and EM9-XH cells. More DNMT1 is cleared from chromatin in XRCC1 complemented cells following 24 hours recovery.

SUPPLEMENTARY Figure 6. Olaparib disrupts co-localization between DNMT1 and XRCC1 foci after a treatment with 5-azadC. The histogram shows the mean and standard deviation of the Manders co-localization index (n=10). The difference is statistically significant according to the Student´s t test P<0.01.

SUPPLEMENTARY Figure 7. Quantification of SSBs induced by 5-azadC in combination with the PARP inhibitor 4-ANI as assessed by the ADU technique. EM9-V (XRCC1 deficient) and EM9-XH (*XRCC1* complemented) cells were treated for 24 hours with increasing concentrations of 5-azadC for 24 hours alone or in combination with 10 µM of 4-ANI during the last 12 hours. The means and SD of three independent experiments are shown.

SUPPLEMENTARY Figure 8. Quantification of SSBs induced by 5-azadC in combination with the PARP inhibitor 4-ANI as assessed by ADU technique. EM9-V (XRCC1 deficient) and EM9-XH (*XRCC1* corrected) cells were treated for 1 hours with increasing concentrations of 5-azadC alone or in combination with 10 µM of 4-ANI. After that, media was changed and cells were allowed to grow for 1 hour in the presence/absence of fresh 4-ANI. The means and SD of two repeats from the same experiment are shown.

SUPPLEMENTARY Figure 9. 5-azadC in combination with Olaparib induces apoptosis in HL60 cells. The AML cell line HL60 was incubated at optimal growth conditions with a combination of 250 nM 5-azadC and 2 µM Olaparib for 12, 24 and 48 hours. The cells were then harvested and stained with Annexin-V FITC/7AAD and analyzed using flow cytometry. These data show an early presentation of phosphatidyl serine residue and later uptake of 7AAD, suggesting death by apoptosis.

SUPPLEMENTARY MATERIAL AND METHODS

Detection of abasic (AP) sites using the ARP probe.

Exponentially growing AA8 and EM9 cells were treated with 5-azadC (50 µM) for 24 hours, harvested, washed in cold PBS, and fixed in cold 70% ethanol for 30 minutes.

After that, cells were processed according to the AP site detection kit from Abcam. This kit uses an Aldehyde Reactive Probe (ARP) which is N’-aminooxymethylcarbonylhydrazino-D-biotin. This ARP has high affinity for abasic sites and is labeled with biotin. Cells were finally incubated with FITC-conjugated avidin and cellular fluorescence was determined with a Beckman Coulter cytomics FC 500 MPL flow cytometer. Fluorescent signals were collected using the standard configuration of the flow cytometer (green fluorescence for FITC). Fluorescence was also observed by microscopy, in this case cultures were treated as indicated and processed according to manufacturer´s instructions (Abcam).

Supplementary Figure 1

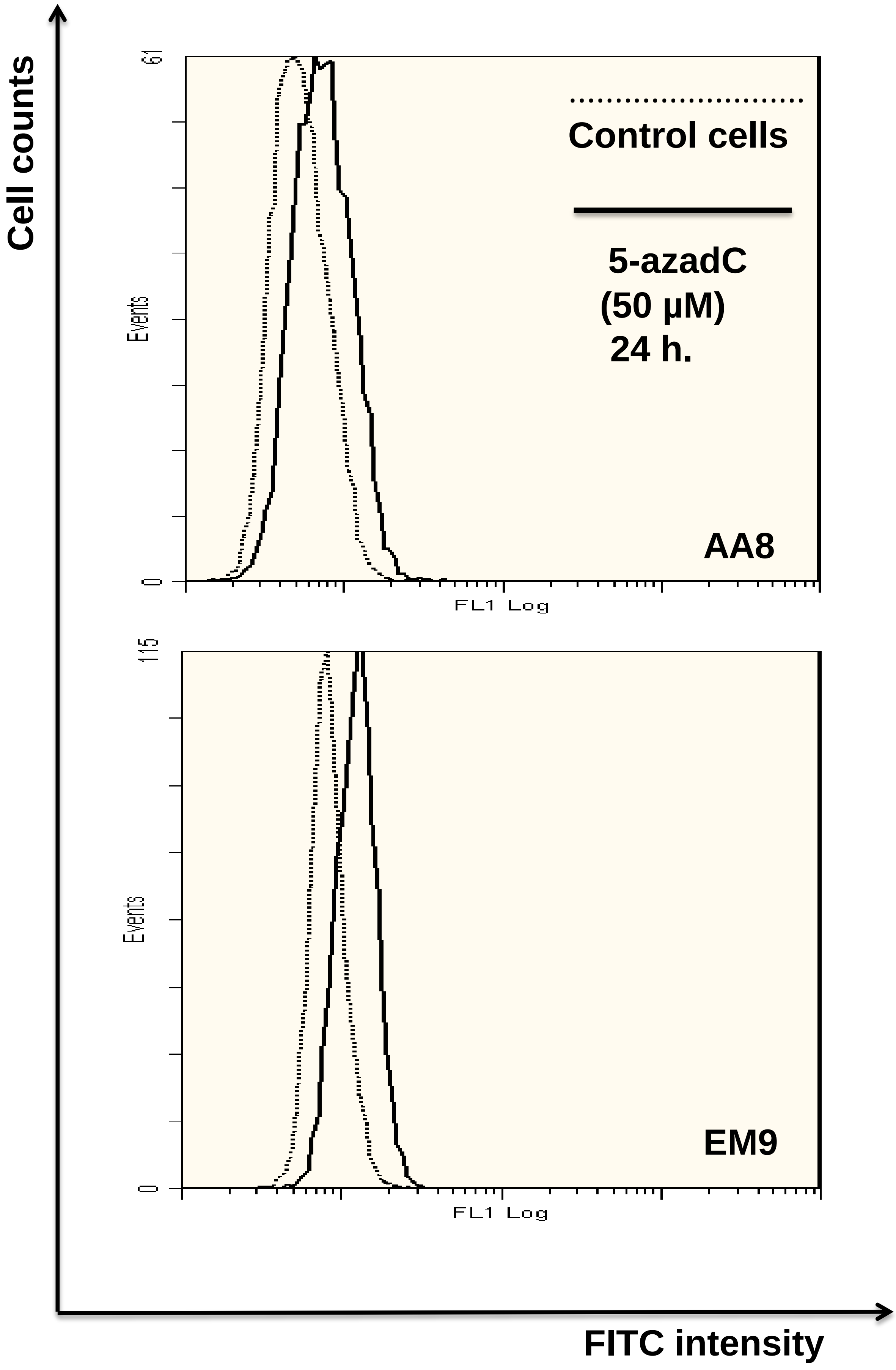

Supplementary Figure 2

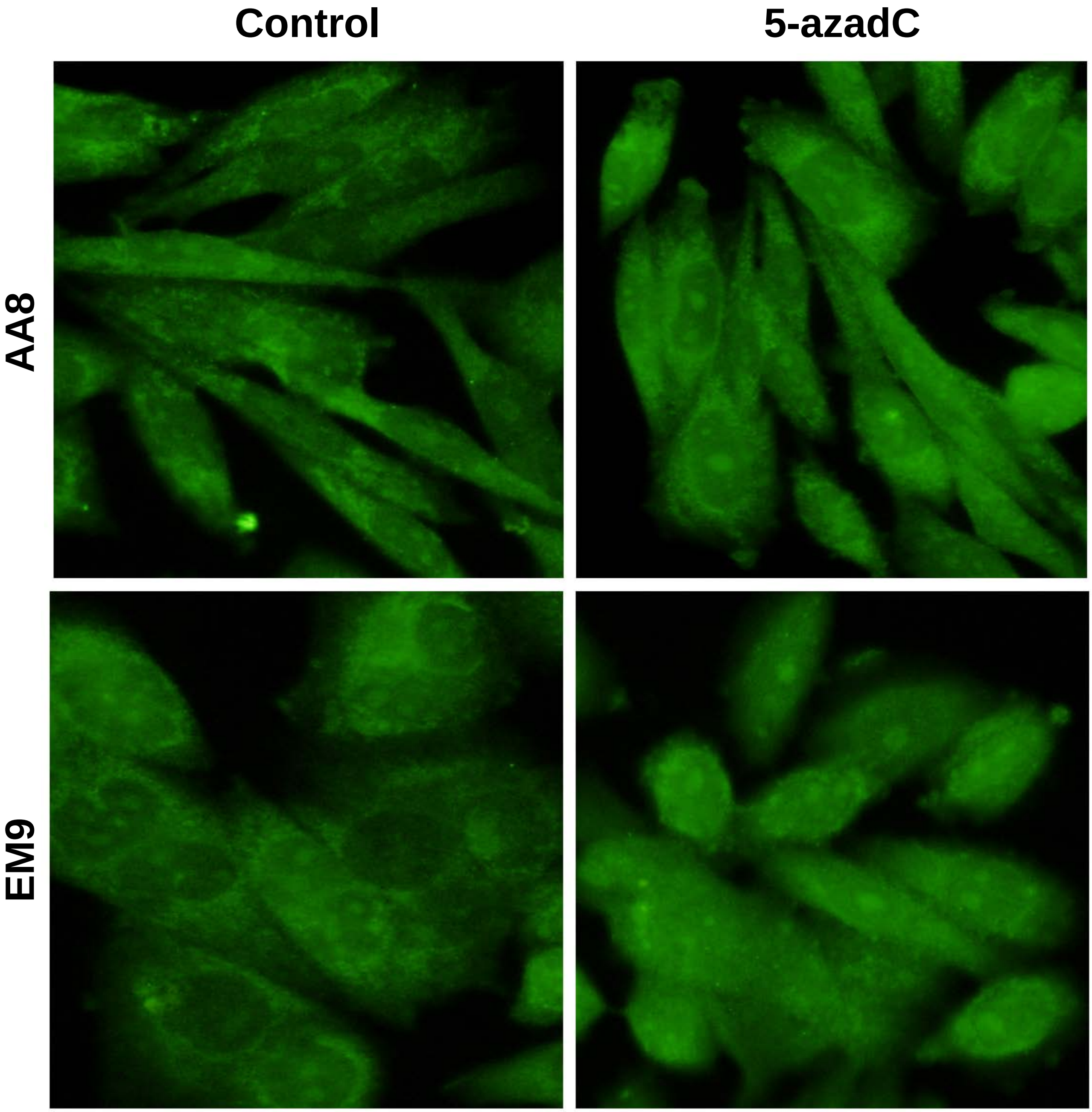

Supplementary Figure 3

A

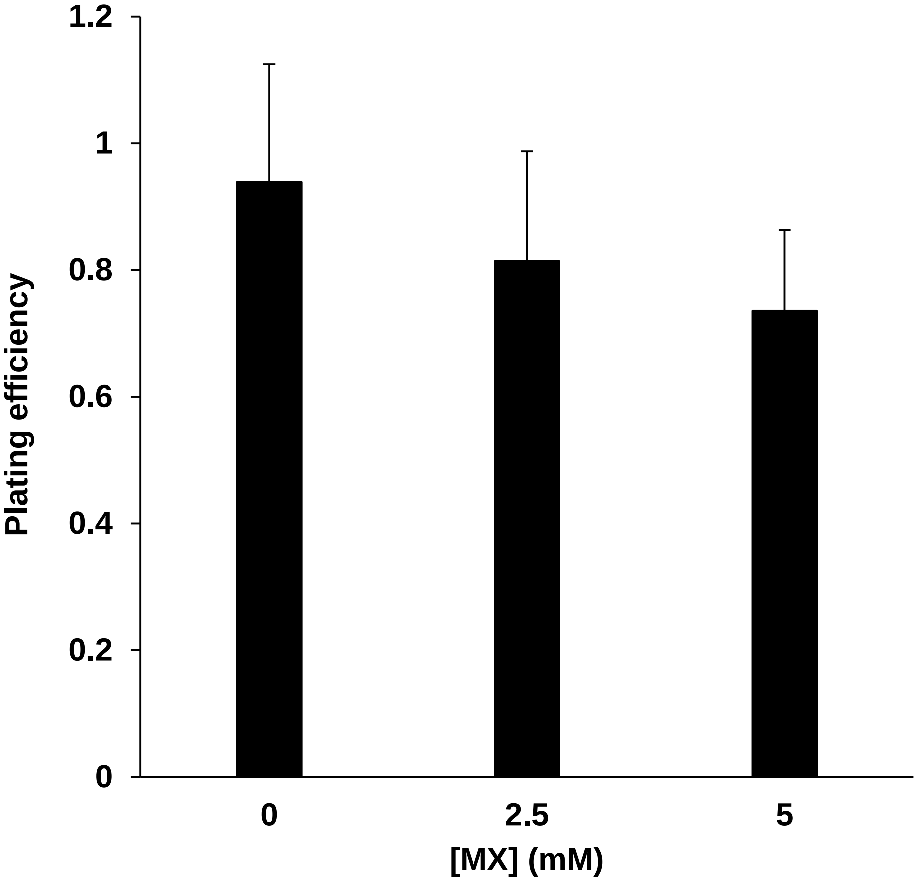

B

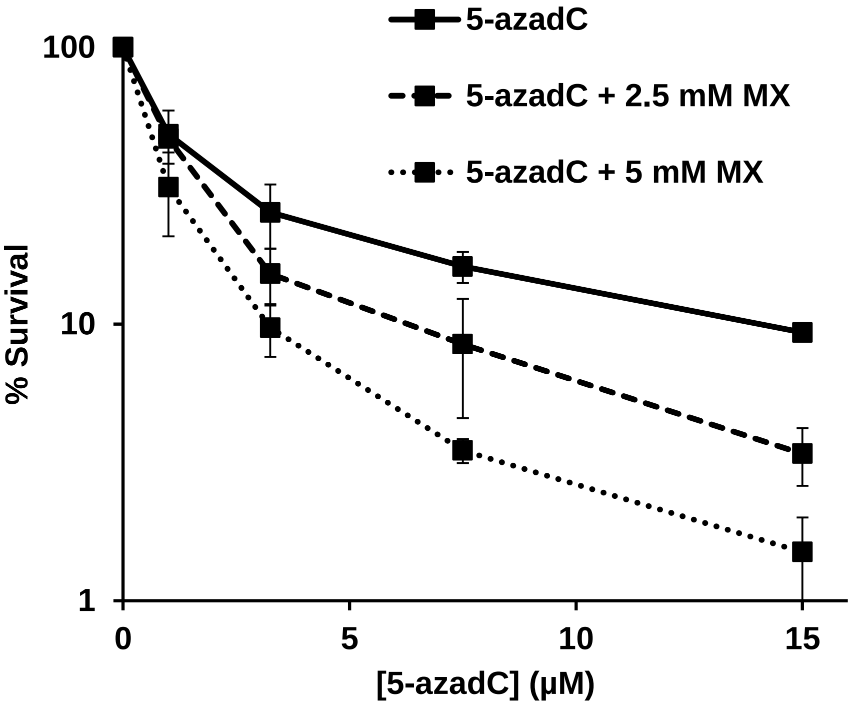

Supplementary Figure 4

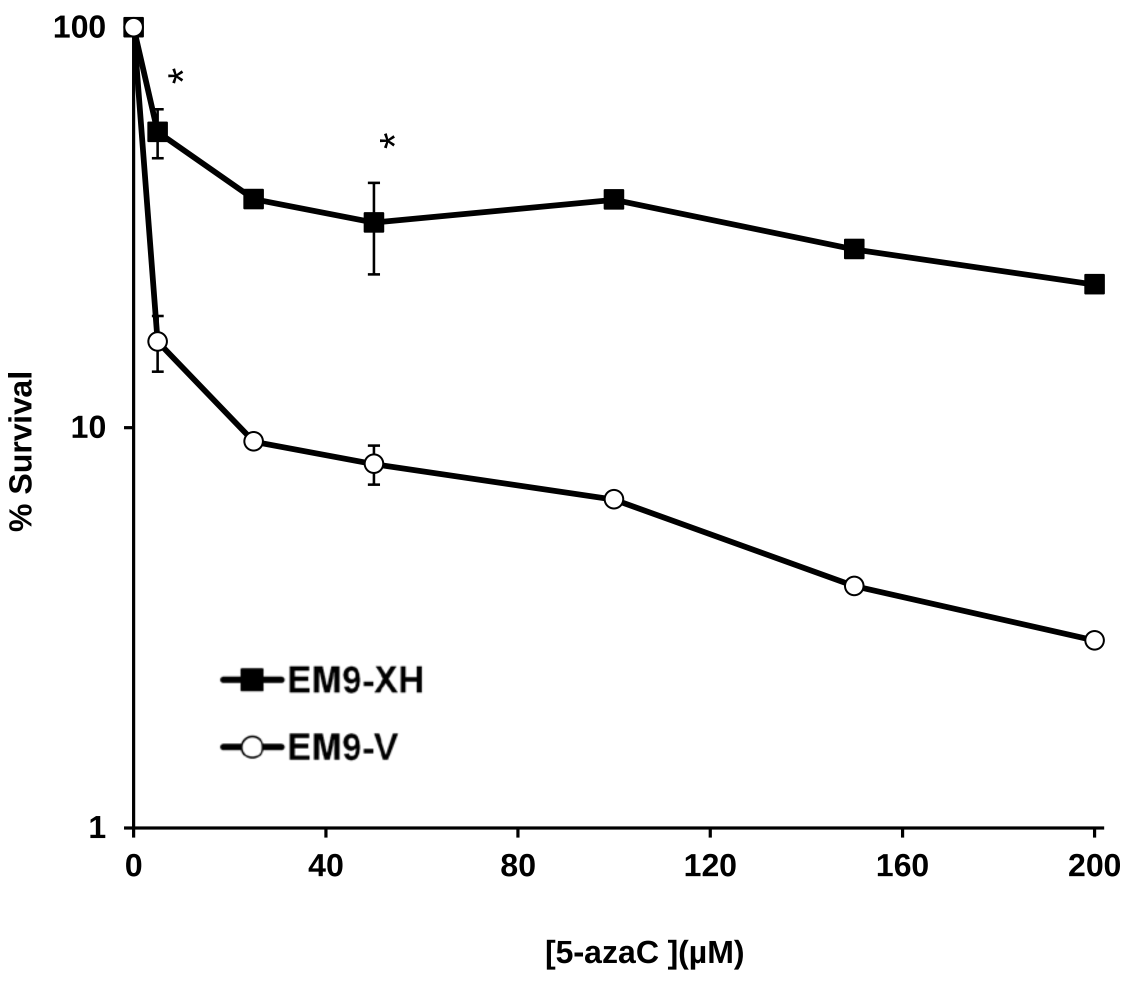

Supplementary Figure 5

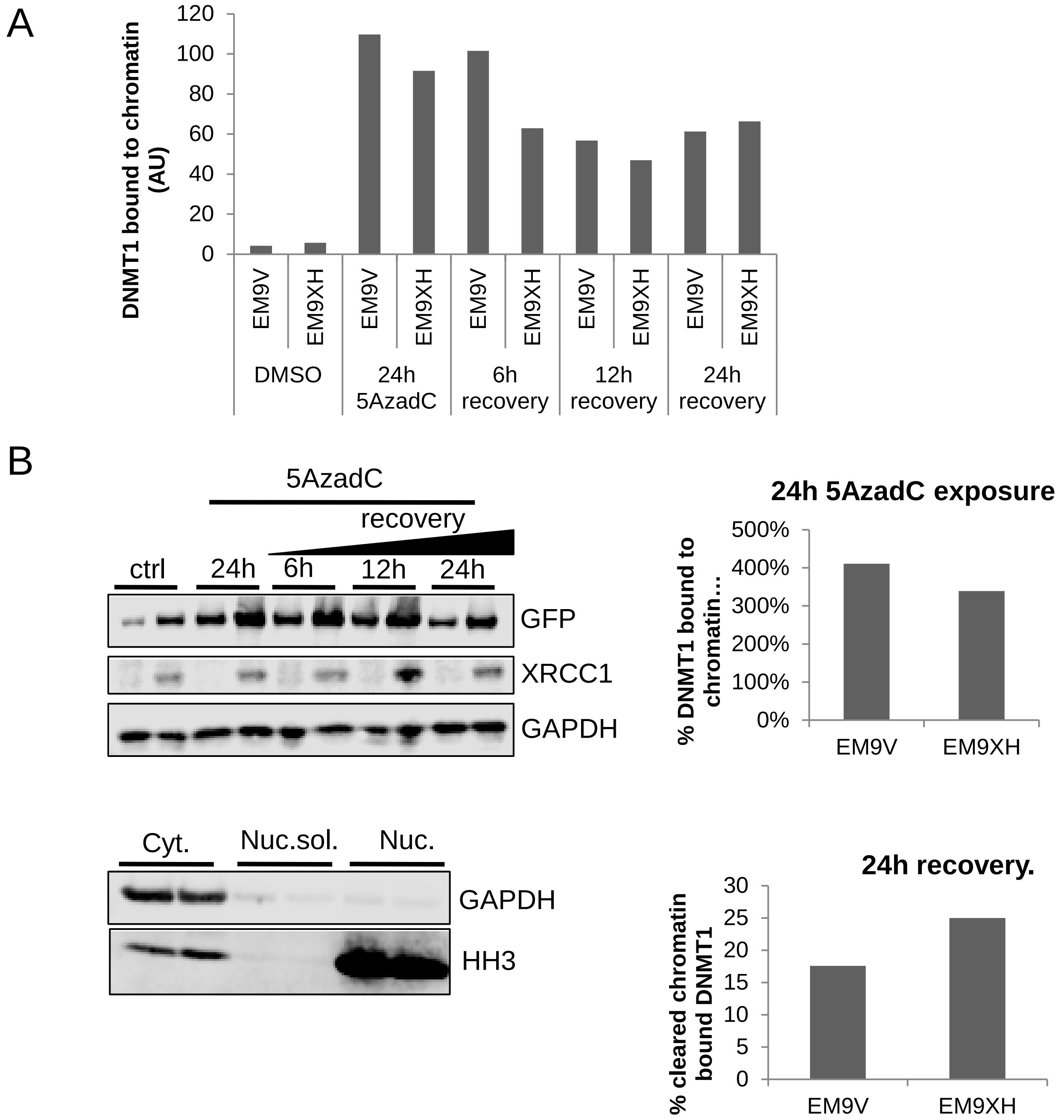

Supplementary Figure 6

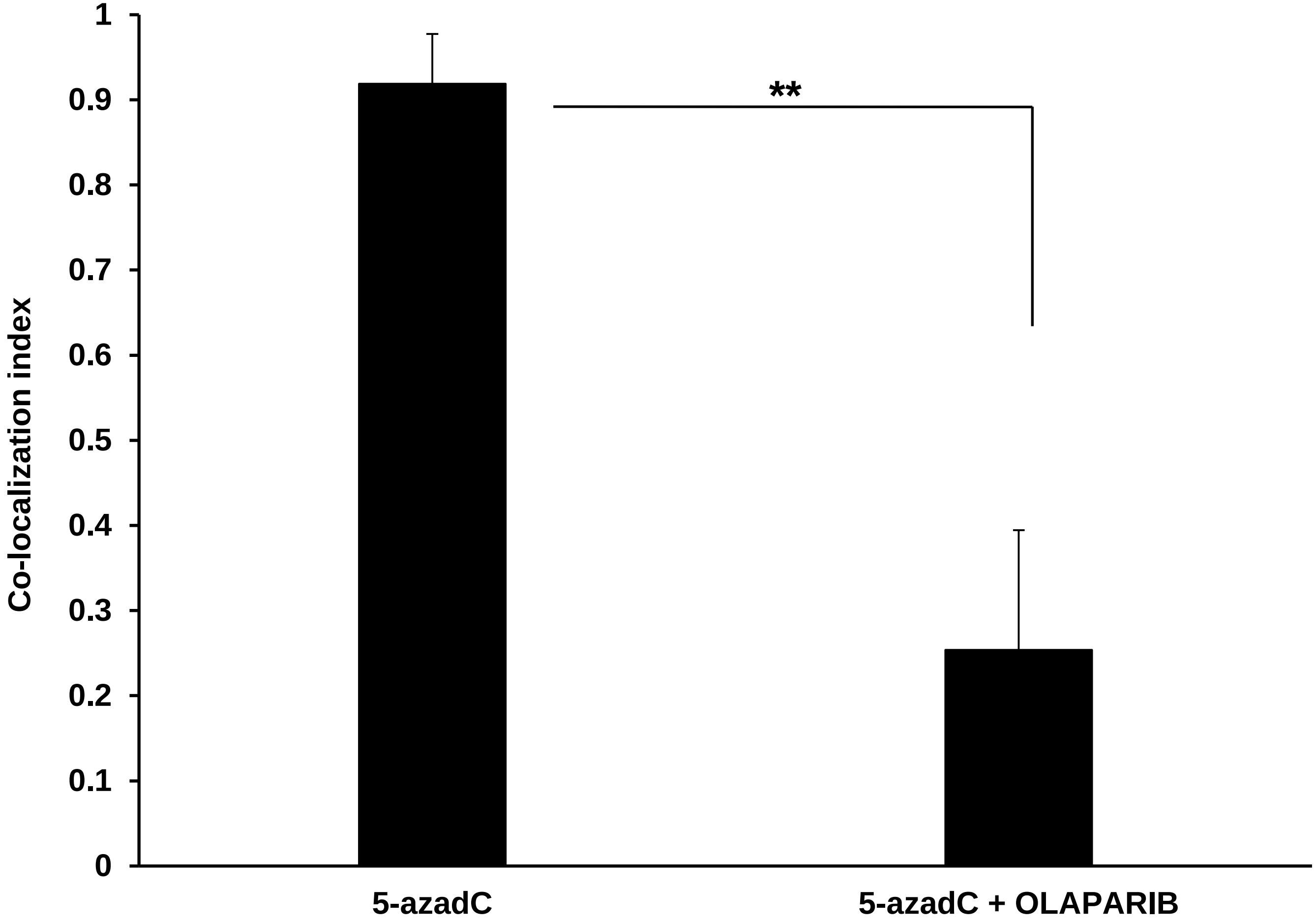

Supplementary Figure 7

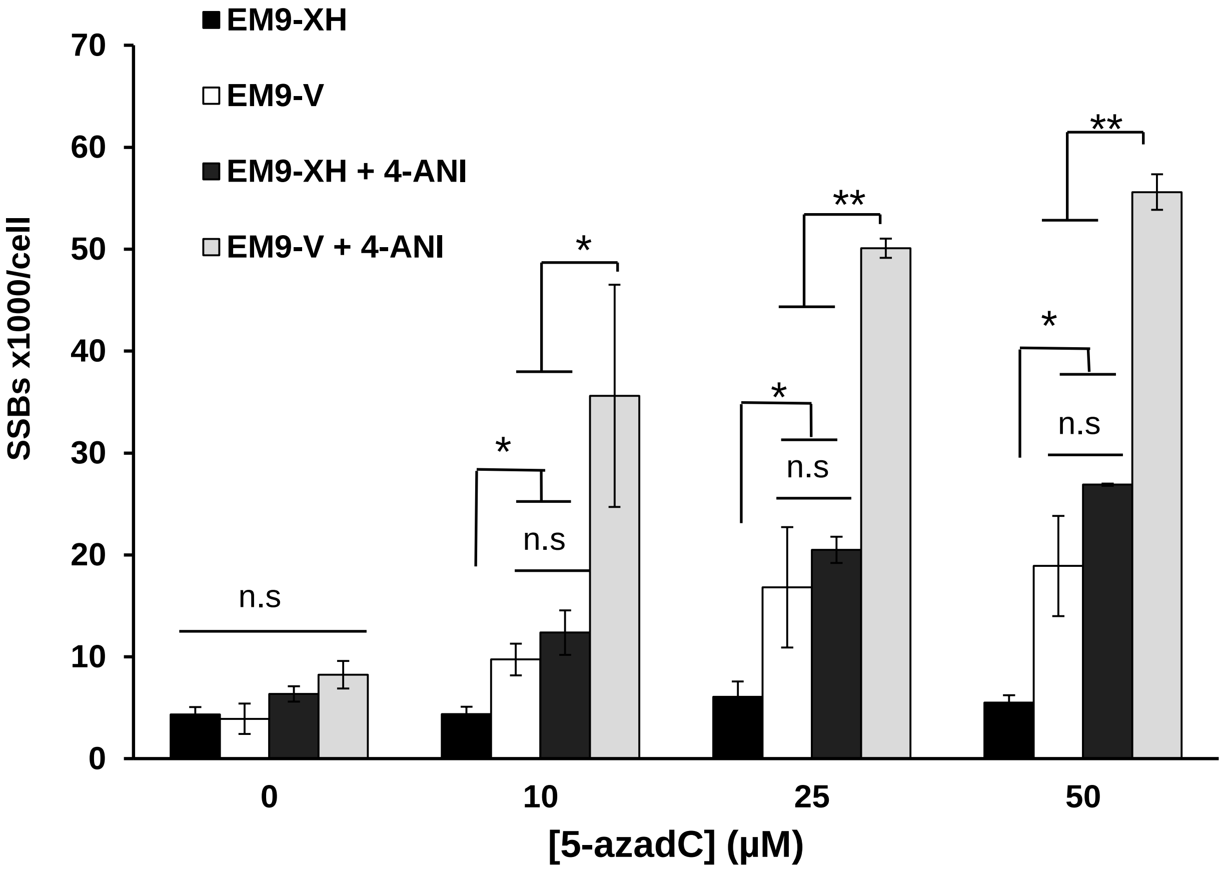

Supplementary Figure 8

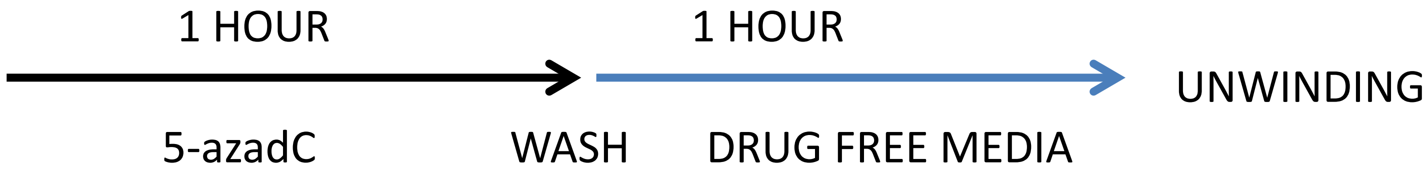

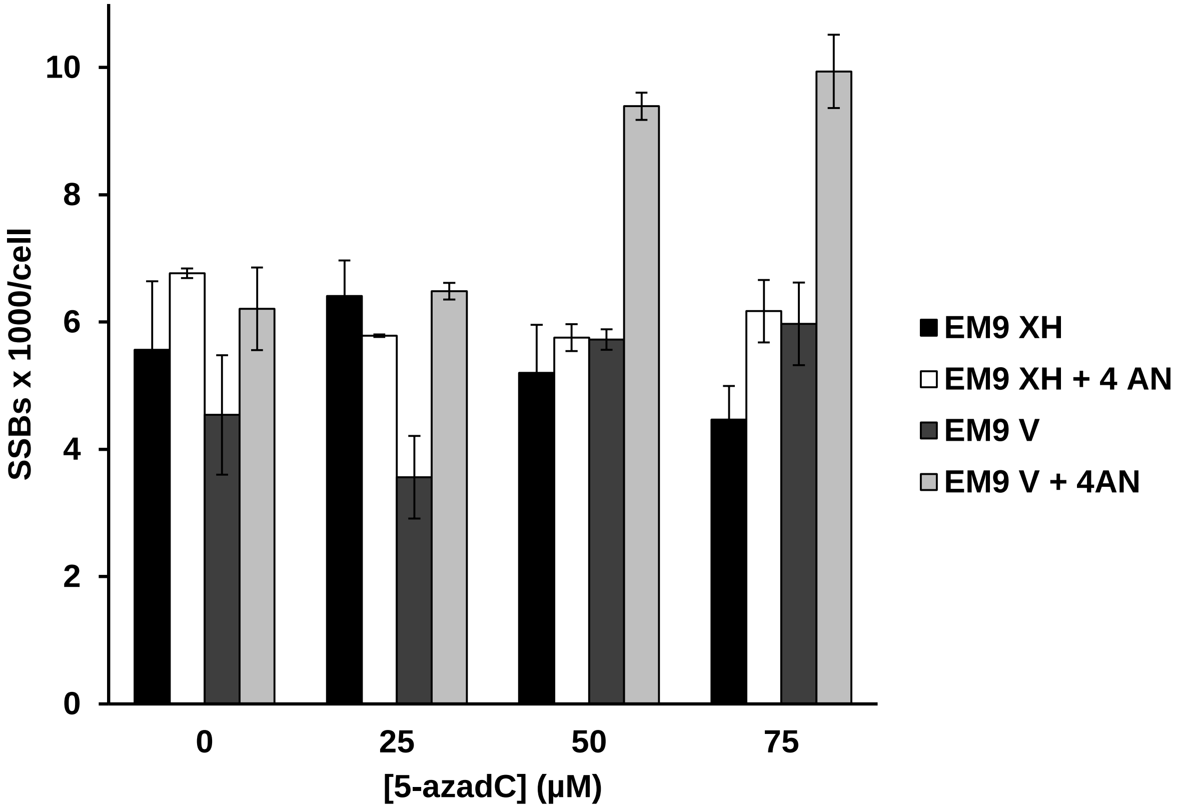

Supplementary Figure 9

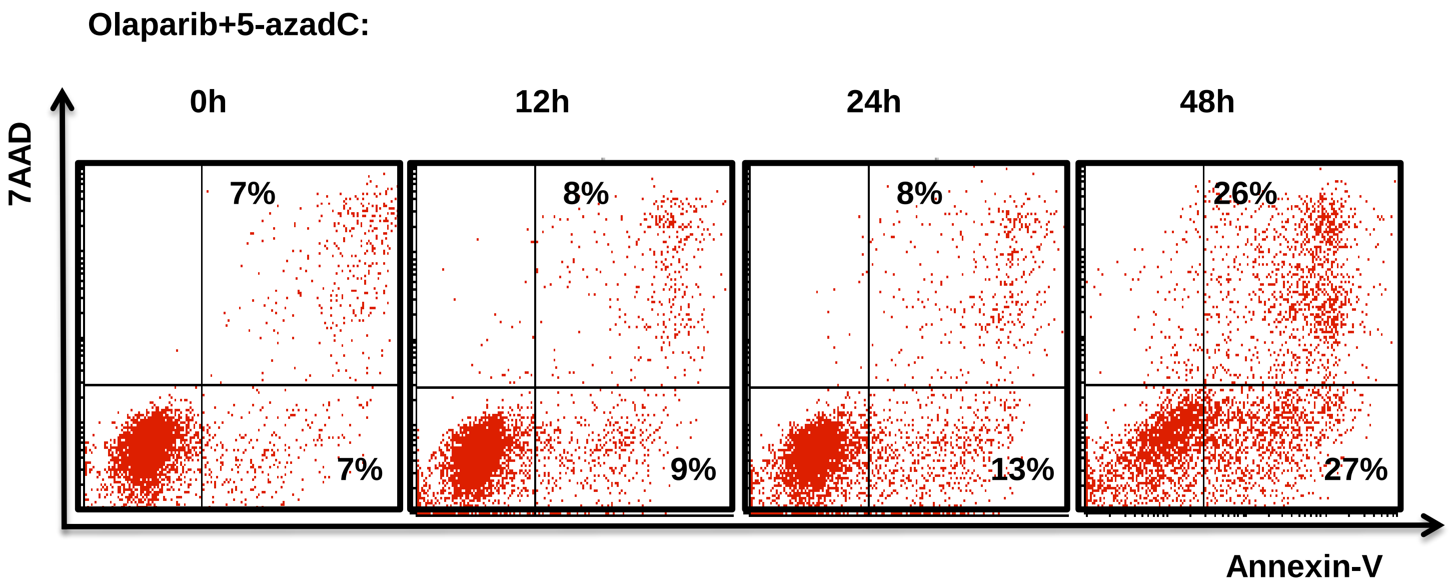

Supplement: SUPPLEMENTARY DATA [file supp_gku638_nar-00391-v-2014-File009.pdf]
